# Variance-corrected Michaelis-Menten equation predicts transient rates of single-enzyme reactions and response times in bacterial gene-regulation

**DOI:** 10.1038/srep17820

**Published:** 2015-12-04

**Authors:** Otto Pulkkinen, Ralf Metzler

**Affiliations:** 1 Department of Physics, Tampere University of Technology, FI-33101 Tampere, Finland; 2 Institute for Physics & Astronomy, University of Potsdam, D-14476 Potsdam-Golm, Germany

## Abstract

Many chemical reactions in biological cells occur at very low concentrations of constituent molecules. Thus, transcriptional gene-regulation is often controlled by poorly expressed transcription-factors, such as *E.coli* lac repressor with few tens of copies. Here we study the effects of inherent concentration fluctuations of substrate-molecules on the seminal Michaelis-Menten scheme of biochemical reactions. We present a universal correction to the Michaelis-Menten equation for the reaction-rates. The relevance and validity of this correction for enzymatic reactions and intracellular gene-regulation is demonstrated. Our analytical theory and simulation results confirm that the proposed variance-corrected Michaelis-Menten equation predicts the rate of reactions with remarkable accuracy even in the presence of large non-equilibrium concentration fluctuations. The major advantage of our approach is that it involves only the mean and variance of the substrate-molecule concentration. Our theory is therefore accessible to experiments and not specific to the exact source of the concentration fluctuations.

The basic question of enzymology concerns the rate of a reaction, in which a substrate-molecule *S* first forms a complex *SE* with an enzyme, and upon catalysis turns into a product *P*. The reaction, commonly written as


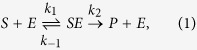


was first described and analysed by Henri^1^,^2^. His work was picked up ten years later by Leonor Michaelis and Maud Leonora Menten, who then presented a thorough derivation and interpretation of the equation^3^


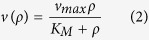


for the reaction-rate *v* as a function of the substrate concentration *ρ*. *K*_*M*_ = (*k*_−1_ + *k*_2_)/*k*_1_ is the Michaelis-Menten constant, and the maximal rate is *v*_*max*_ = *k*_2_ × [*E*], where [*E*] is the enzyme concentration. Equation (2) is commonly known as the Michaelis-Menten equation (MME).

The derivation of the MME relies on a series of assumptions. First, the step in which the SE complex is turned into a product, is in general reversible. This can lead to ‘blocking’ of the reaction pathway by products turning back into substrate-molecules, or even activate the reaction if the enzyme has several conformational forms^4^,^5^. However, if the products are immediately removed from the system by some other reaction, or their concentration is otherwise small, the backward reaction can be neglected. The second major assumption in the derivation of the MME is that of a quasi-steady state, which says that the concentration of the complex *SE* in the reaction scheme (1) does not change considerably on the time scale of the product formation^6^. Alternatively, one may assume fast equilibration of the complex with the free substrate, which leads to a slightly different form of *K*_*M*_ = *k*_−1_/*k*_1_ called the dissociation constant in this case. The validity of the quasi-steady state approximation has been discussed, for instance, by Rao and Arkin^7^.

Third, and perhaps most fundamentally, the whole derivation of the MME is based on deterministic ordinary differential equations, in which the total substrate and enzyme concentrations enter as parameters. In practice, this means that the solution of constituent molecules must be well-mixed by fast diffusion to avoid local concentration differences. Moreover, there must be large numbers of constituent particles in a large reaction volume in order to be able to define concentrations and to neglect the fluctuations due to the inherently stochastic nature of the reaction and substrate import or synthesis. The breakdown of the MME due to the finiteness of the reaction volume were predicted and discussed by Grima^8^. Also, the effects of small enzyme numbers on the reaction have been under theoretical study since the 1960’s^9^, yet the statistics of the turnover times of single enzymes were thoroughly analysed^10^ and confirmed in the laboratory only within the past decade^11^,^12^. Theoretical results for rates of single-enzyme reactions were reviewed in ref. 13.

In this article, we show that concentration fluctuations due to stochastic substrate production and degradation can drastically change the reaction-rate from the prediction of Eq. (2), especially in the particular problem of gene-regulation by highly specific transcription-factors. Previously, Stéfanini, McKane and Newman^14^ solved a model with input of single substrate-molecules into a small cellular compartment which acts as the reaction volume. Their formula for the reaction-rate is remarkable because it is a very simple function of the mean compartmental substrate concentration alone, even if the process effects a non-zero variance. This is probably due to the Poissonian nature of the input process in their model. Already in the 1970’s, Smeach and Smith^15^ had presented a solution to a more complicated model with Poissonian removal of substrate-molecules. The solution is much more complicated than the one by Stéfanini *et al.*, and cannot be expressed solely in terms of the mean substrate concentration. Effects of substrate concentration fluctuations on turnover times of single-enzyme molecules were discussed, for instance, in refs 16,17. However, no explicit, universal correction to the Michaelis-Menten formula due to substrate fluctuations appears to exist in the literature. In the next section, we derive mathematically exact upper and lower bounds for the reaction-rates of enzymatic reactions under such fluctuations and propose a first order correction to the Michaelis-Menten equation. As we will demonstrate, this variance correction works exceptionally well.

## Results

### Variance-corrected Michaelis-Menten equation

It is a quite common feature that the concentrations of molecules that are genetically expressed change in time in an abrupt manner: The concentration can be almost constant for a long period of time, although the cell growth causes some gradual change. Occasionally the concentration jumps by a significant amount due to a translational (and/or transcriptional) burst or cell division^18^,^19^,^20^,^21^,^22^,^23^,^24^. This behavior contrasts the slow concentration drifts and only small fluctuations around the mean when there is a large number of molecules present in a reaction volume such as a biological the cell. For sufficiently large concentrations, the rate of a reaction involving these molecules as a substrate follows the MME (2) with the constant concentration *ρ* replaced by the (time or ensemble averaged) mean 〈*ρ*(*t*)〉 of a stochastic, time dependent concentration *ρ*(*t*). On the other hand, in case of abrupt and relatively large concentration changes, as in bursty protein production, the reaction-rate *v*(*ρ*(*t*)), defined as the number of reactions per unit of time, depends on the exact concentration *ρ*(*t*) in each cell and therefore shows high cell-to-cell variability. More precisely, out of an experiment with a large sample of cells, the number of reactions that have occurred up to time *t* for each cell and the rate computed from that information exhibits large cell-to-cell variations. The quantity that describes the occurrence of the reaction on the population level, and hence also provides the best estimate for the reaction rate in a single cell, is the *ensemble average* 〈*v*(*ρ*(*t*))〉, *i.e.* the rate averaged over all cells. Next we show that this estimate can be significantly lower than the naive approximation *v*(〈*ρ*(*t*)〉) based alone on the mean substrate concentration in the population.

The starting point for our analysis is the observation that the MME (2) is a concave function of the concentration *ρ*. In terms of the normalised reaction-rate *v*_0_ = *v*/*v*_*max*_, we obtain by Jensen’s inequality^25^ for concave functions that


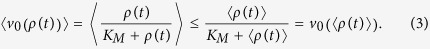


This means that the MME (2) provides an upper bound for the reaction rate for fluctuating substrate concentrations. Conversely, evaluating the remainder in the integral form^26^ of Taylor’s theorem,


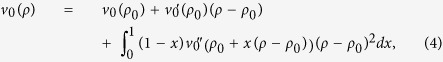


with *ρ*_0_ = 〈*ρ*(*t*)〉, and after ensemble averaging we find


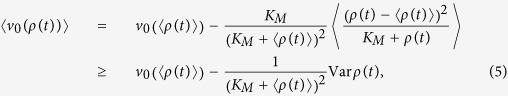


where Var*ρ*(*t*) = 〈(*ρ*(*t*) − 〈*ρ*(*t*)〉)^2^〉 is the variance of the concentration *ρ*(*t*), and the last inequality follows from *ρ*(*t*) ≥ 0. The inequality is clearly the sharpest that can be expressed as a function of the mean and variance of the substrate concentration alone. Any further refinement requires the knowledge of higher moments of the concentration distribution. As the MME (2) sets the optimal upper bound, given deterministic dynamics, we have thereby accurately bounded the effects of stochastic substrate fluctuations on the rate of the Michaelis-Menten reaction scheme (1) from both above and below. The bounding of the Michaelis-Menten reaction-rate is our first important result.

The natural candidate for a refined equation for the reaction-rate is the one given by a second order Taylor approximation for small fluctuations around the mean. We call this the variance-corrected Michaelis-Menten equation (VCMME);


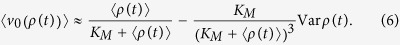


**Figure 1:**
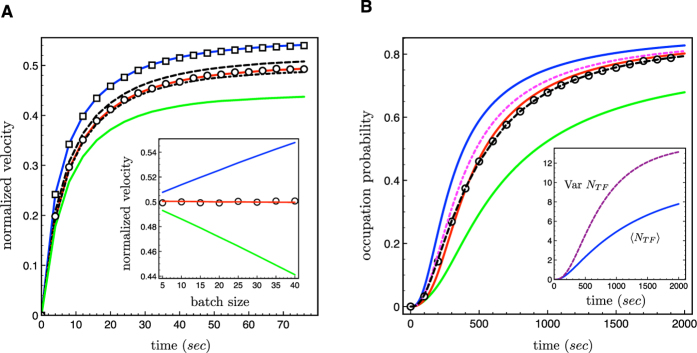
Stochastic corrections to rates of enzymatic reactions and efficiency of transcriptional gene-regulation. (**A**) Normalised rate *v*_0_ of a single-enzyme reaction as a function of time for bursty substrate production. The system is initially void of substrate-molecules. They are produced in batches of size 40 with the production times determined by a Poisson process, and there are on average 117.6 molecules present in the stationary state. The variance-corrected MME (red solid line) coincides almost exactly with the simulation results (circles). The uppermost, blue solid line is the Michaelis-Menten upper bound based on the mean density alone, and the lowermost, green solid line shows the optimal lower bound. The figure also shows the third (dashed) and fourth (dash-dot) order Taylor approximation to 〈*v*_0_〉. Squares are the solution of Stefanini *et al.*^14^ for the system with batch size 1. Inset: Predictions by the usual MME and optimal lower bound deviate linearly from the variance-corrected MME and simulation results with increasing batch size. (**B**) The occupation probability *p*_0_ of the TF-Operator complex as a function of time in a non-stationary gene regulation experiment. The uppermost, blue solid line is the Michaelis-Menten upper bound based on the mean density alone, and the lowermost, green solid line shows the optimal lower bound. The variance-corrected MME (red solid line) yields an excellent approximaxxtion to the results (12)–(18) of the analytical model (dashed line) and the simulation results (circles). The pink dash-dotted line is the Poissonian approximation given by Eq. (19). Inset: The density (blue solid line) and population variance (purple dashed line) of the number of TF molecules in a single cell as a function of time.

It has the desired properties of reducing to the MME in the deterministic limit and falling between the optimal variance bounds for all values of the mean concentration, its variance and the constant *K*_*M*_. In fact, the corrected equation (6) attains the mean value of the optimal upper and lower bounds at the mean concentration 〈*ρ*(*t*)〉 = *K*_*M*_, which marks the point at which the usual MME reaches half of its maximum and the enzymes are in the substrate-bound complex state approximately half of the time. Figure 1A shows that the variance-corrected approximation is able to predict the rate of a reaction catalysed by a single enzyme in a cellular compartment with bursty input of substrate-molecules: The only difference to the usual reaction scheme (1) in this example is that the substrate-molecules enter the reaction volume in batches. With growing batch size, the concentration fluctuations increase and the contribution of the variance correction in Eq. (6) becomes more significant.

There are clear reasons why we truncate the Taylor series at the term proportional to the variance: First, the truncated form is experimentally accessible. One only needs to measure the substrate concentration variance in addition to the mean concentration. Second, the full Taylor series of the ensemble-averaged reaction rate


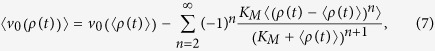


is typically an alternating series (that is, successive terms have opposite signs) because the right tail of the concentration distribution is longer and also possibly heavier at low mean substrate concentrations in cellular environments. It turns out that the third order approximation (series truncated so that the last term is *n* = 3) and fourth order approximation (*n* = 4) can yield poorer results than the series truncated at the variance correction (*n* = 2), as indicated by the simulation results of Fig. 1A. In fact, nothing guarantees that the rate of convergence of the Taylor series is fast, and a very high order approximation might be needed to outperform the VCMME. In such cases, most of the higher order terms eventually cancel. We conjecture that only if the fluctuations are very large compared to the mean concentration and *K*_*M*_, or if the concentration distribution is highly skewed, higher order corrections are needed to get an accurate estimate for the reaction-rate. This point is further discussed below in an application to gene-regulation.

The VCMME works equally well for a system of multiple enzymes, although the resulting higher rate of production and removal of substrate from the system must be compensated by an increased rate of substrate import, which reduces the relative strength of stochastic effects. Also, the formula does not differentiate between different sources of noise that show up in the variance. It is quite remarkable that the accuracy of the new formula is insensitive to such details. It is even more surprising in the light of the fact that the fluctuations of the substrate concentration depend on the reaction-rate itself. This is because the reaction is, in absence of cell growth, cell division and substrate efflux, the only pathway of substrate concentration dilution. Exact evaluation of the reaction-rate would therefore involve solving a closed system of Kolmogorov equations as pursued, for instance, by Smeach and Smith^15^ and Stéfanini *et al.*^14^ in the case of substrate input one molecule at a time. The assumption of quasi-equilibrium permits bypassing the feedback loop and directly taking the average of the reaction-rates over a distribution of substrate concentrations. The VCMEE and the demonstration of its remarkably accuracy compared to fully stochastic simulations is the central result of this work.

### Application to gene-regulation

**Figure 2:**
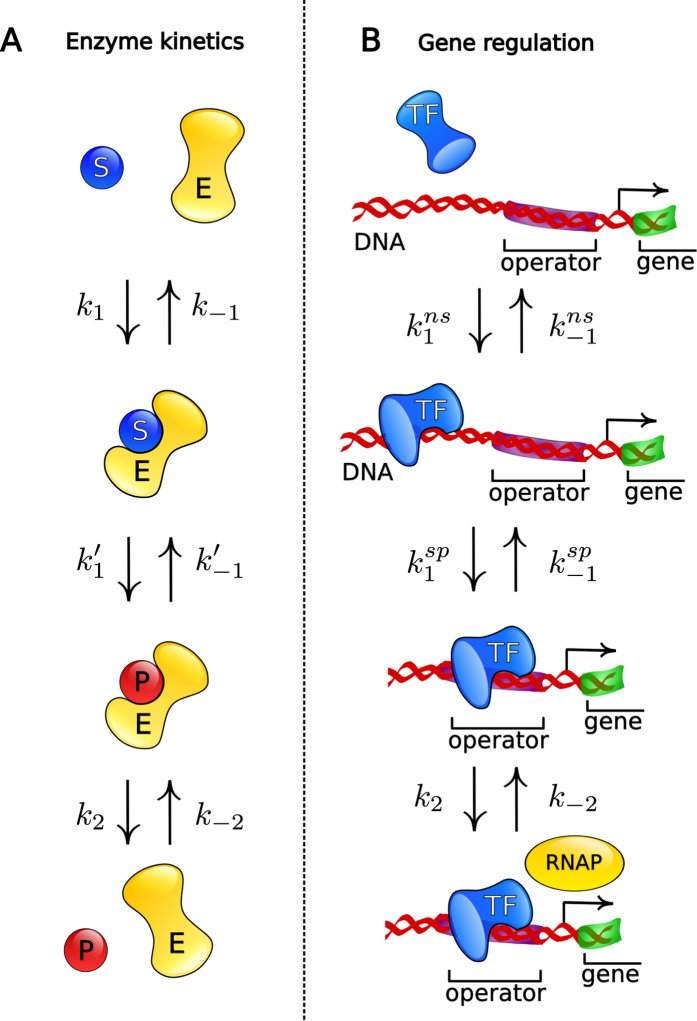
Similarities and differences in enzyme kinetics and transcriptional gene-regulation. (**A**) In an enzymatic reaction, substrate S and enzyme E form a complex SE, which turns into a complex PE of product and enzyme. The product P is released in the final step. As a consequence, the number of substrate-molecules in the reservoir is decreased by one. (**B**) In transcriptional gene-regulation, a TF molecule first binds the DNA non-specifically and finds its specific binding site, the operator O, by sliding along the DNA. The operator bound TF changes the DNA conformation or interacts with RNA polymerase (RNAP) directly, which changes the transcription rate of the target gene. The TF molecule is returned to the reservoir of free TF upon unbinding from the DNA, and therefore the total number of TF is conserved. The probability of the complex states (third step) both in enzyme kinetics and gene-regulation is described by a Michaelis-Menten type equation as discussed in the main text.

Transcription-factors (TFs) find their specific binding sites on the DNA by facilitated diffusion: They diffuse in the cytoplasm and, upon contact, bind non-specifically to the DNA and slide along it and simultaneously probe the nucleotide sequence^27^,^28^,^29^,^30^. The enhancement of the stochastic search of TFs for their specific binding site on the DNA was shown to be relevant in living cells^31^,^32^,^33^,^34^. As shown in Fig. 2, this process of non-specific binding of a TF to the DNA and consequent specific binding to the operator is similar to the formation of a substrate-enzyme complex and its transformation to a complex of a product and enzyme. More precisely, the kinetics of a TF within a reaction distance for non-specific binding to the DNA around the target corresponds to an augmented Michaelis-Menten reaction without release of a product (see the reaction scheme (1))


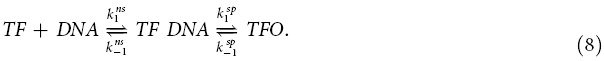


Here *TF DNA* represents the non-specifically DNA-bound TF and *TFO* is the TF-operator complex.

As the TF slides along the DNA, the rate of interconversion between the non-specific and specific binding modes of a TF must be very high (*e.g.* order of 10^6^/*s* for *LacI*^27^) for the TF to recognize its binding site as it diffuses over it. Hence we assume equilibrium of the non-specifically bound state. Denoting the equilibrium constants for non-specific and specific binding by 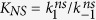 and 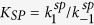, respectively, the occupation probability *p*_0_ of the operator as a function of the TF concentration *ρ*(*t*) around the target can then be shown to obey a Michaelis-Menten type formula^35^


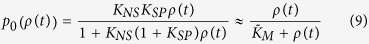


where 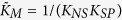 and the last approximation is justified if the binding at the operator is strong, that is, *K*_*SP*_ ≫ 1. This is typically fulfilled for TFs^36^,^37^,^38^,^39^. We adopt this approximation in the following analysis, yet the cases with smaller *K*_*SP*_ can be treated in a similar manner. We believe that the mapping of the TF binding process onto the Michaelis-Menten scheme is an important concept in the study of molecular signalling in biological cells.

In order to obtain a formula for the mean occupancy 〈*p*_0_(*ρ*(*t*))〉 of the operator, we need the statistics of the TF concentration *ρ*(*t*), and for that a realistic stochastic model for the expression of the TF. We include the necessary stochastic steps in the TF production, given by the coupled reactions


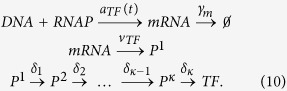


The first line describes the synthesis of mRNA as the RNA polymerase binds to the DNA, followed by the degradation of individual mRNA molecules by Ribonuclease E. The second line represents translation of mRNA to polypeptide chains of amino acids by ribosomes. The third line is the protein maturation process, which involves *κ* separate steps, such as formation of the nucleus and secondary and tertiary structures in protein folding. Each step in the diagram is modelled as a Poisson process with the indicated rates. The sum of the random lag times Δ_*i*_ = 1/*δ*_*i*_ in protein maturation is denoted by Δ.

The above process determines the number of mature TF molecules produced on a given time interval. This data still needs to be converted to the concentration *ρ* of TF molecules available for binding at the target site, and the effects of cell growth and division, as well as a possible efflux of molecules, have to be included. We set


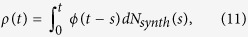


where *ϕ*(*t*) = *ϕ*(***x***_*TF*_, ***x***_*O*_, *t*) is the concentration of TFs in the reaction volume surrounding the operator (located at ***x***_*O*_) of the transcription unit given that a mature TF molecule emerged at location **x**_*TF*_ at time zero. In particular, causality requires *ϕ*(*t*) = 0 for *t* < 0. The stochastic process *N*_*synth*_ marks the TF synthesis times according to the reaction scheme (10). In mathematical terms, *N*_*synth*_ is a Poisson point process with a stochastic varying intensity, the events of which are each independently delayed by a random amount Δ. More precisely, the intensity of the Poisson process *N*_*synth*_ is itself a random process *v*_*TF*_*N*_*mRNA*_(*t*), *i.e.*, the product of translation rate and the number of mRNA molecules in the cell. The latter evolves according to a *birth-death process*^40^ with mRNA synthesis rate *a*_*TF*_(*t*) (independent of the system state) and degradation rate *γ*_*m*_ for each mRNA molecule. As for the enzymatic reactions discussed in the previous section, we only consider the simplest time dependence of the transcription rate *a*_*TF*_(*t*) of the TF gene, namely a zero rate for *t* < 0 and a constant rate *a*_*TF*_ > 0 for *t* ≥ 0. This implies the initial condition *N*_*mRNA*_(0) = 0 for the number of TF transcripts. The reason for choosing this particular scenario is that this way we can directly observe the response of the target gene to a change in the transcription rate of its regulator.

The statistics of the TF available for binding can be solved exactly within the theoretical framework explained above. This can be achieved by inspecting the generating function for the master equation^41^ or, alternatively, by using the Laplace transform 〈exp(−*λρ*)〉 of the TF concentration. The latter is convenient in our case because the probability (9) of specific binding at the target can be easily obtained by integration,


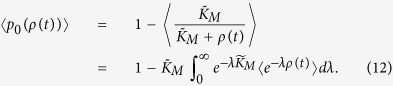


Given the definition (11) of the TF concentration we first observe that the delay Δ = Δ_1_ + … + Δ_*κ*_ for each point of the process *N*_*synth*_ has the distribution^42^


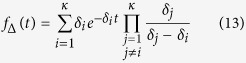


for *t* ≥ 0 and zero otherwise. Furthermore, the Laplace transform can be written in the form (see SI for full derivation)


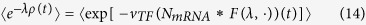



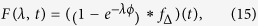


with the asterisk denoting convolution 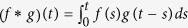 starting at time zero. The angular brackets on the right-hand side of Eq. (14) denote the ensemble average over trajectories of the birth-death process *N*_*mRNA*_ for mRNA synthesis. According to diagram (10), the process has a state-independent birth rate *a*_*TF*_ and death rate *γ*_*m*_ per molecule. We show in SI that the convolution functional of Eq. (14) can be calculated analytically even for a general integrable function of time in place of *F*(*λ*, *t*) and therefore also for a general physical distribution *ϕ*(***x***_*TF*_, ***x***_*O*_, *t*) of TF molecules in Eq. (11). Our final result for the Laplace transform is


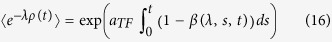


where


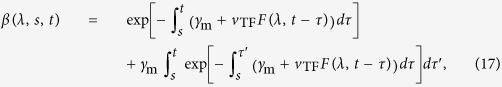


and *F* is as defined in Eq. (15). The probability (12) of specific TF binding at the target can now be computed by substituting the exact form of the Laplace functional and integrating the resulting expression numerically. Formulae (15), (16) and (17) together form another important result.

At this point, we concentrate on the stochastic effects of small molecular copy numbers in the cell, and hence leave the implications of the nucleoid and other structures^43^,^44^,^45^,^46^,^47^,^48^, observed in *E. coli* and other bacteria, as a subject of further study. Therefore we take the TF transport to be a fast diffusive process in a homogeneous environment, which can be modelled by setting


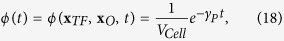


where *γ*_*P*_ is the rate of protein (in this case the TF) dilution due to efflux, cell growth and division, and *V*_*Cell*_ is the average cell volume. In particular, the function *ϕ* does not depend on the location of the TF gene or the binding site in the cell, whereas in a more detailed model it would be a solution of a space-inhomogeneous diffusion equation^35^,^49^. Moreover, protein partition upon cell division is modelled by constant dilution of the TF concentration. Explicit inclusion of cell division events would increase the TF variability in the time trajectories from individual cells^20^, and although the consequent variability is of different type than in gene expression noise, its effect can be small on a population level^24^. Simulations on our system show that the protein partitioning already averages out to a large degree in ensembles that include data from hundreds of cells (see SI).

Figure 1B shows that for biologically realistic parameters the analytical results (12)–(18) are in excellent agreement with results from simulations using the Gillespie algorithm. The VCMME provides a very good approximation to the exact result, whereas the error in the prediction for the probability of specific binding given by the ordinary MME can be as large as 0.15 and even larger for higher, plausible TF translation rates (see SI for parameter sweeps). Moreover, the difference is significant for the whole duration of the transient, extending to half an hour in the example considered, but this time period can be even longer in real cells with slowed-down TF transport and cell division effects. These results remain valid even if there are multiple TF binding sites on the DNA (see SI). We also observe from Fig. 1B that the VCMME performs better than an analytical model based on simple Poissonian fluctuations of the number of TF molecules in the cell, in which case the occupation probability reads


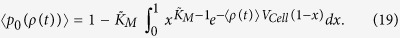


The last result means that it can be more important to include all sources of noise in the first stochastic correction in the VCMME rather than try to come up with an approximative model for which all the central moments can be computed.

Figure 1B also shows that the curve for the binding probability is a sigmoidal function of time—even in the case of the ordinary MME, which itself is a non-sigmoidal function of the TF concentration by definition. Consequently, the initial slow rise must come from the time evolution of the mean concentration, caused by the equilibration of the transcription process and by protein maturation delays. To this end, let us study the analytical formula for the mean TF concentration,


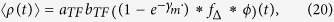


obtained by differentiating the Laplace transform (16) with respect to *λ* and taking the limit *λ* → 0. The new constant *b*_*TF*_ = *v*_*TF*_/*γ*_*m*_ stands for the mean size of a translational burst. Eq. (20) shows that the mean concentration is a convolution of three terms, each of which represents a different process in the reaction diagram (10): The exponential term describes the equilibration of the number of transcripts, *f*_Δ_ protein maturation, and *ϕ* the distribution and dilution of mature TF. A concave function of time is recovered only in the limit of infinitely fast translation and mRNA degradation (in such a way that *b*_*TF*_ approaches a non-zero constant) in the absence of maturation delays. With delays, a simple calculation using definition (20) shows that the initial rate of growth is


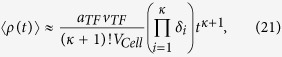


so each stochastic intermediate step in protein synthesis increases the exponent of time by one. Result (21) can be used to determine the number *κ* of intermediate states in the maturation process. The stationary mean concentration equals *a*_*TF*_*b*_*TF*_/(*γ*_*P*_*V*) as expected^19^.

Similarly, the variance of the TF concentration can be obtained from the second derivative of the Laplace transform (16). However, it is not expressible as a simple convolution but takes the form


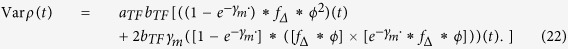


This is also a sigmoidal function of time—it only reduces to the known concave limit as the maturation delays vanish and translational bursts are instantaneous, that is, *γ*_*m*_, *v*_*TF*_ → ∞. The slight difference with the result of refs 19,21, as the homogenous diffusion kernel (18) is inserted, originates from the dilution of proteins instead of explicit degradation. The graphs of the mean concentration and variance as functions of time are depicted in the inset of Fig. 1.

**Figure 3:**
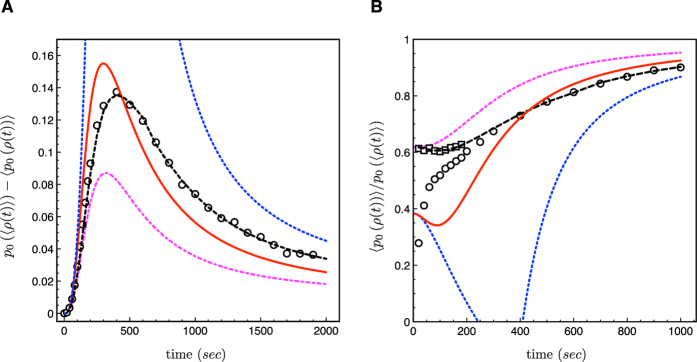
Sources and magnitude of stochastic corrections in transcriptional regulation. (**A**) The curves show the difference of the Michaelis-Menten prediction and various stochastic refinements for the probability of specific binding at the operator. Circles are the Monte Carlo simulation data, the black dashed line is the analytical model, the magenta dash-dotted line is the Poisson approximation, and the red solid line is the VCMME (6). (**B**) Relative correction 〈*p*_0_(*ρ*)〉/*p*_0_(〈*ρ*〉). The colour and line type coding is the same as in the left panel. Additionally, the squares are results of a Monte Carlo simulation in which the TF is able to bind to the DNA in both nonspecific and specific forms independently. In that case, the TF-DNA complex is close to equilibrium even at small times. Below 200 seconds, the Monte Carlo simulation data of the full model differ significantly from the analytical and numerical results of the model that assumes equilibrium of the TF-DNA intermediate state.

Figure 3A shows that the error in the MME prediction for the occupation probability is the largest at intermediate times. The curves for the difference *p*_0_(〈*ρ*(*t*)〉) −* *〈*p*_0_(*ρ*(*t*))〉 share the same qualitative features in the full analytical, variance-corrected and Poissonian models: The initial increase is well approximated by 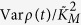, *i.e.* it is proportional to the variance, as shown by the blue dashed line. The magnitude of the correction reaches its maximum around 300 to 400 seconds, and finally, it approaches a stationary value at rate 
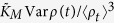
. The final value can be estimated from the stationary mean and variance of the TF concentration using the VCMME. In the stationary state, the variance of the TF concentration is proportional to the square of the translational burst size. Since the stationary mean protein level is not affected by the length of translation bursts, as long as the burst size is constant, whereas the variance grows as the bursts get shorter, the limit of instantaneous translation bursts provides an upper bound for the magnitude of the variance correction to the MME. The maturation delays also slightly increase the stationary variance—the effect being the strongest for *κ* = 1*, i.e.* for a single maturation step. However, since the stationary correction is small in comparison to the transient correction, we associate the particularly strong deviation from the deterministic theory with non-equilibrium fluctuations in the TF concentration, which arise at short and intermediate times.

We stress that the TF concentration fluctuations are clearly the main reason for the difference of the deterministic and stochastic occupation probabilities. In particular, the assumption of fast equilibration of the TF-DNA complex seems to have little or no effect at all. This is confirmed by the close match of the analytical (assuming equilibrium) and simulation (no equilibrium assumption made) results in Fig. 3A. The breakdown of an intermediate equilibrium can only be seen at the shortest times by inspecting the ratio 〈*p*_0_(*ρ*(*t*))〉/*p*_0_(〈*ρ*(*t*)〉). In particular, the plot in Fig. 3B reveals that at the very beginning of the experiment and up to 200 seconds, when the TF concentration is still very small, the analytical model, the VCMME, and the Monte Carlo simulation all exhibit different behavior: The ratios from the analytical model and from the VCMME start at constant levels at time zero, and have a minimum around 100 seconds, whereas the stochastic simulation data increases monotonically. Extrapolation to the beginning of the experiment suggests that the initial value in the simulation is close to zero, implying a significant deviation from the deterministic dynamics. Figure 3B also shows that the results of the analytical model match the data from another Monte Carlo simulation, in which the TF is able to bind to the DNA independently in both the non-specific and specific forms, and binding kinetics therefore equilibrates much faster. Hence, we associate the observed deviation to the breakdown of the intermediate state equilibrium. The description of the TF binding dynamics to DNA in terms of the VCMME relevant for typical *in vivo* concentrations is our other central result.

## Discussion

We demonstrated that stochastic concentration fluctuations can lead to a significant correction to the Michaelis-Menten equation of reaction kinetics. The proposed first order correction is conceptually simple and experimentally accessible—it only requires the ensemble variance of the fluctuating molecule concentration. Yet it turns out to be highly accurate according to our analytical and numerical computations with detailed models of catalysed chemical reactions and transcriptional gene-regulation. In the wake of massive experimental advances allowing single molecular insight into signalling processes in living cells^50^,^51^ we believe that our results are both timely and relevant for a more accurate description of reactions at typical *in vivo* concentrations.

Interestingly, a correction term to the MME resembling the one we propose, but of a completely different origin, was derived in ref. 52. In that analysis, a second term in the MME emerges as a consequence of differentiable changes in *deterministic* substrate concentrations, and the only stochasticity in the problem lies in the kinetics of the catalysis. A connection to non-equilibrium quantum mechanical geometric phases was suggested. We believe that the similarity of the two theories can be explained by the theory of linear response: The geometric phase correction term to MME is proportional to the time derivative 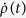, which probably can be expressed in terms of concentration fluctuations due to dilution of substrate by the stochastic enzymatic reaction. However, the variance correction to MME can be used to embrace other sources of stochasticity as well—in particular upstream fluctuations, and it is not restricted to small fluctuations and thus represents a more general and flexible concept.

It was recently suggested that the fluctuations of intermediate metabolite concentrations in network structures consisting of multiple enzymatic processes are typically uncorrelated^53^. This would mean that spontaneous fluctuations do not spread out in the network. In mathematical literature, this is known to be a general feature of so called quasi-reversible stochastic networks, for which a Poisson process input into the network implies a Poisson process output^54^. While this might be true in networks consisting of enzymatic reactions only, we have seen that gene-regulation networks are more susceptible to upstream fluctuations. In particular, operator state fluctuations can determine much of the global state of the network. Therefore, it is important to extend the study of fluctuation propagation to larger scale networks using the corrections presented here. This also requires a study of multimerisation and cooperativity because the Hill equation is not necessarily concave in those cases, and the rate at mean concentration is no longer an upper bound for the true reaction-rate^55^. However, the second order Taylor approximation is still the natural candidate for a corrected equation of promoter activity in those cases.

Upstream fluctuations in substrate concentration and the kinetics of substrate binding are not the only stochastic components in the reaction kinetics. For example, conformational changes of enzymes^5^,^10^,^16^,^56^ may also affect the reaction-rate. A recent study also pointed out that the rate of an enzymatic reaction is sensitive to the form of the distribution for the unbinding times of the substrate from the enzyme^57^. For example, in some cases, increasing the substrate concentration only decreases the rate. Such unbinding distributions could be a consequence of anomalous conformational fluctuations of substrate-molecules and enzymes, as observed experimentally for single proteins^58^,^59^. The joint contribution of both conformational and concentration fluctuations will potentially lead to unexpected phenomena in reaction kinetics and needs to be thoroughly investigated.

We are confident that our results will inspire a series of new studies of the detailed reaction mechanisms in enzymatic and gene-regulatory reactions at typically small concentrations both in living cells and the extracellular environment. A particularly pressing question is how such reactions are modified in view of recent studies demonstrating the anomalous diffusion of transcription factor-size green fluorescent proteins (GFPs) in the cytoplasm and nucleoplasm of eukaryotic cells^60^ as well as in the presence of superdiffusive, active mixing^61^.

## Methods

All Monte Carlo simulations presented in this study were performed using the Gillespie algorithm^62^. The numerical integration of the analytical results was implemented in Mathematica. The full list of parameters used in producing the figures, as well as additional figures with higher transcription and translation rates for the TF in gene-regulation, can be found in the SI Appendix. Derivation of formula 14 for the Laplace transform of the TF concentration and a general formula for exponential functionals of immigration-death processes, which leads to result (16), is also provided in the SI Appendix.

## Additional Information

**How to cite this article**: Pulkkinen, O. and Metzler, R. Variance-corrected Michaelis-Menten equation predicts transient rates of single-enzyme reactions and response times in bacterial gene-regulation. *Sci. Rep.* **5**, 17820; doi: 10.1038/srep17820 (2015).

## Supplementary Material

Supplementary Information
