# Supplementary material for: Variance-corrected Michaelis-Menten equation predicts transient rates of single-enzyme reactions and response times in bacterial gene-regulation
Source: Sci Rep. 2015 Dec 4;5:17820. doi: 10.1038/srep17820 (PMC4669464; doi:10.1038/srep17820)
Supplement: Supplementary Information [file srep17820-s1.pdf]

Supplementary Information:
Variance-corrected Michaelis-Menten equation predicts transient rates of single-enzyme reactions and response times in bacterial gene-regulation

Otto Pulkkinen[1] and Ralf Metzler[2,1]

[1]*Department of Physics, Tampere University of Technology, FI-33101 Tampere, Finland*
[2]*Institute for Physics & Astronomy, University of Potsdam, D-14476 Potsdam-Golm, Germany*

I. SI APPENDIX 1: SOLVING THE DETAILED MODEL OF GENE REGULATION

In this Appendix, we present mathematical derivations which are too long to be included in the main text. We adopt some new notation from mathematical probability for the Appendix: Here, $\mathbb{E}f$ stands for the mathematical expectation of f in the sense of integrating f with respect to the joint probability distribution of all random variables present in it. For long expressions, this is more convenient than the bracket notation. Moreover, $\mathbb{E}[f|\mathcal{F}]$ will denote the conditional expectation of f given information $\mathcal{F}$ because there is no fixed notation for conditional expectations in physics and chemistry nomenclature (in particular, the one using a vertical line between the brackets is easily confused with an inner product).

A. Step 1: Inclusion of protein maturation delays

According to definition in the main text, the TF concentration at the target site equals

$$\rho(t) = \int_0^t \phi(t-s)\, dN_{synth}(s) \tag{1}$$

where $\phi(t) = \phi(\mathbf{x}_{TF}, \mathbf{x}_O, t)$ is the TF concentration in the reaction volume surrounding the operator (located at $\mathbf{x}_O$) of the transcription unit given that a single mature TF molecule emerged at location $\mathbf{x}_{TF}$ at time zero. $N_{synth}(t)$ is the point process in time describing protein synthesis, that is, of TF molecules, at location $\mathbf{x}_{TF}$. In the following derivation, we do not presume any particular form of ϕ (although spatially homogenous form is assumed in the simulations presented in the main text and further Appendices). Hence, this Appendix provides a mathematical result for a very general class of models of reaction kinetics in spatially structured environments. For the current application in mind and for notational simplicity, however, we shall omit the the spatial indices in function ϕ.

The Laplace transform of the TF density can be written as

$$\mathbb{E}e^{-\lambda\rho(t)} = \mathbb{E}\exp\left(-\lambda\int_0^t \phi(t-s)\, dN_{synth}(s)\right) = \mathbb{E}\exp\left(-\lambda\sum_{i=1}^{N_{synth}[0,t)} \phi(t-\tau_i)\right), \tag{2}$$

where $0 < \tau_1 < \ldots < \tau_{N_{synth}[0,t)} < t$ are the synthesis times of mature TF molecules, and $N_{synth}[0,t)$ is the total number of mature molecules produced on the time interval $[0,t)$. For $N_{synth}[0,t) = 0$, the sum in the exponential of Eq. [2] is empty. Each synthesis time can be further split as a sum of time of synthesis of the nascent polypeptide chain by a ribosome τ_i^0 and a protein maturation delay Δ_i such that $\tau_i = \tau_i^0 + \Delta_i$. As explained in the main text, each Δ_i is a sum of κ exponential random variates with parameters δ_i, $i = 1,\ldots,\kappa$, whence its probability distribution function reads

$$f_\Delta(t) = \sum_{i=1}^{\kappa} \delta_i e^{-\delta_i t} \prod_{\substack{j=1 \\ j\neq i}}^{\kappa} \frac{\delta_j}{\delta_j - \delta_i}\,. \tag{3}$$

In order to compute the expectation over τ_i^0 and Δ_i in Eq. (2), we use the following trick: The time interval $[0,t)$ is split into n consecutive, disjoint intervals of equal length such that $[0,t) = \cup_{j=0}^{n-1}[t_j, t_{j+1})$ with $t_0 = 0$. On each of these intervals we assume

1. The number of mRNA is constant: $N_{mRNA}(t) = N_{mRNA,j} := N_{mRNA}(t_j)$ for $t \in [t_j, t_{j+1})$.
2. Each nascent polypeptide produced within the same interval experiences exactly the same value of the stochastic maturation delay $\Delta^{(j)}$ which itself is distributed the same way as the original delays. That is, $\Delta_i = \Delta^{(j)}$ almost surely for all i such that $\tau_i^0 \in [t_j, t_{j+1})$.

The first assumption is not very restrictive because N_{mRNA} is a piecewise constant birth-death process, which will be recovered (in a suitable topology [1]) as we eventually send the number of intervals $n \to \infty$. The second may seem strange at first because each nascent molecule is supposed to go through *a different* stochastic realization of the maturation pathway with varying time duration. However, here we use the fact that the points of Poisson processes are mutually exclusive in the sense that for a (almost surely) finite rate $v_{TF} N_{mRNA}(t)$, each interval will for large enough n contain at most one point, *i.e.* translation event. Hence all maturation delays will be independent and identically distributed according to the law of the original Δ_i's in the limit $n \to \infty$, and we can safely take this limit at the end of the calculation.

Let $\tau^0_{i,j}$ be the time at which the ith translation event that takes place on the jth interval $[t_j, t_{j+1})$, and let $\Delta_{i,j}$ be the delay associated with it. Let further $N^0_{synth}[t_j, t_{j+1})$ denote the total number of translation events $\tau^0_{i,j}$ on the indicated interval, and let $U[t_j, t_{j+1})$ denote a uniformly distributed random variable on it. For the moment, we consider the translation rate $v_{TF} N_{mRNA}(s)$, $s \in [0, t)$ as a known function (so we are actually calculating a conditional expectation with complete information on the trajectory of $N_{mRNA}(s)$), and we will evaluate the average over its paths in the next section. Then

$$
\begin{aligned}
&\mathbb{E} \exp\left(-\lambda \sum_{i=1}^{N_{synth}[0,t)} \phi(t-\tau_i)\right) = \mathbb{E} \exp\left(-\lambda \sum_{i=1}^{N^0_{synth}[0,t)} \phi(t-\tau^0_i - \Delta_i)\right) \\
&= \mathbb{E} \exp\left(-\lambda \sum_{j=0}^{n-1} \sum_{i=1}^{N^0_{synth}[t_j,t_{j+1})} \phi(t-\tau^0_{i,j} - \Delta_{i,j})\right) = \prod_{j=0}^{n-1} \mathbb{E} \prod_{i=1}^{N^0_{synth}[t_j,t_{j+1})} \exp\left(-\lambda \phi(t-\tau^0_{i,j} - \Delta_{i,j})\right) \\
&\approx \prod_{j=0}^{n-1} \mathbb{E} \exp\left(-\lambda \phi(t - U[t_j,t_{j+1}) - \Delta^{(j)}) N^0_{synth}[t_j,t_{j+1})\right) \\
&= \prod_{j=0}^{n-1} \mathbb{E} \left(\frac{1}{t_{j+1}-t_j} \int_{t_j}^{t_{j+1}} \int_0^\infty e^{-\lambda\phi(t-s-\delta)} f_\Delta(\delta)\, d\delta\, ds\right)^{N^0_{synth}[t_j,t_{j+1})} \\
&= \prod_{j=0}^{n-1} \exp\left[-v_{TF} N_{mRNA,j} \cdot (t_{j+1}-t_j) \left(1 - \frac{1}{t_{j+1}-t_j} \int_{t_j}^{t_{j+1}} \int_0^\infty e^{-\lambda\phi(t-s-\delta)} f_\Delta(\delta)\, d\delta\, ds\right)\right] \\
&= \prod_{j=0}^{n-1} \exp\left[-v_{TF} N_{mRNA,j} \int_{t_j}^{t_{j+1}} \int_0^\infty \left(1 - e^{-\lambda\phi(t-s-\delta)}\right) F_\Delta(\delta)\, d\delta\, ds\right] \\
&= \exp\left[-v_{TF} \sum_{j=0}^{n-1} N_{mRNA,j} \int_{t_j}^{t_{j+1}} \int_0^\infty \left(1 - e^{-\lambda\phi(t-s-\delta)}\right) f_\Delta(\delta)\, d\delta\, ds\right] \\
&\xrightarrow{n\to\infty} \exp\left[-v_{TF} \int_0^t N_{mRNA}(s) \int_0^{t-s} \left(1 - e^{-\lambda\phi(t-s-\delta)}\right) f_\Delta(\delta)\, d\delta\, ds\right] \\
&= \exp\left[-v_{TF} \left(N_{mRNA} * F(\lambda,\cdot)\right)(t)\right]
\end{aligned}
$$

with $F(\lambda, t) = \left((1-e^{-\lambda\phi}) * f_\Delta\right)(t)$. The last line is the result presented in the main text. The first equality follows from the fact that there are no translation events prior to time zero, and those TFs that mature after the observation time t can be safely included in the calculation because $\phi(t) = 0$ for $t < 0$. The second line comes from the division of the whole time interval into subintervals, and on the third line, the approximation explained above is made. The uniform placement of translation events comes about because of assumption (1), constant translation rate within a subinterval, and the variable $\Delta^{(j)}$ because of assumption (2), the equality of delays for all translation events within a subinterval. The expectation over these two variables is taken on the fourth line, and over the Poisson distributed number of translation events on the fifth. The rest is merely rearrangement of the expression, and finally, taking the limit $n \to \infty$, yields the desired result.

B. Step 2: Inclusion of TF mRNA numbers: formula for exponential functionals of immigration-death processes

To finish the calculation of the Laplace transform of the TF concentration, we need to compute the functional

$$\mathbb{E}\exp\left(-v_{TF}\int_0^t N_{mRNA}(s)F(\lambda,t-s)ds\right)\,, \tag{4}$$

where the exact form of F is given above and in the main text. This is slightly more general than functionals of type $\mathbb{E}\exp\left(-w\int_0^t N(s)\right)$, $w>0$, considered by Picard [2] in a purely mathematical context. Therefore we present the full calculation here. The derivation is valid for a general, positive integrable function F. The number of mRNA, N_{mRNA}, evolves according to a birth-death process with state-independent birth rate $a_T F$, and death rate γ_m for each individual. Due to state independent input rate, these processes are sometimes referred to as immigration-death processes. In the spirit of the calculation of maturation delays, we first consider a piecewise constant approximation such that $F(\lambda,t-s)=F_i:=F(\lambda,t-t_i)$ for all $s\in[t_i,t_{i+1})=\left[\frac{i}{n}t,\frac{i+1}{n}t\right)$, where $i=0,1,\ldots,n-1$. In particular, F (and the function ϕ in our application) can take different values on at most n separate intervals. Introducing the shorthand $A_i=v_{\mathrm{TF}}F_{i-1}\int_{t_{i-1}}^{t_i}N_{mRNA}(s)ds$ for each interval, functional (4) can then be written as a nested integral

$$\begin{aligned}\mathbb{E}\exp\left(-\sum_{i=1}^n A_i\right) &= \mathbb{E}\prod_{i=1}^n e^{-A_i}=\mathbb{E}\left(\prod_{i=1}^{n-1}e^{-A_i}\right)\mathbb{E}\left[e^{-A_n}|\mathcal{F}_{n-1}\right]\\ &=\mathbb{E}\left(\prod_{i=1}^{n-2}e^{-A_i}\right)\mathbb{E}\left[e^{-A_{n-1}}\mathbb{E}\left[e^{-A_n}|\mathcal{F}_{n-1}\right]|\mathcal{F}_{n-2}\right]\\ &=\ldots=\mathbb{E}e^{-A_1}\mathbb{E}\left[e^{-A_2}\mathbb{E}\left[e^{-A_3}\cdots\mathbb{E}\left[e^{-A_{n-1}}\mathbb{E}\left[e^{-A_n}|\mathcal{F}_{n-1}\right]|\mathcal{F}_{n-2}\right]\cdots|\mathcal{F}_2\right]|\mathcal{F}_1\right]\end{aligned} \tag{5}$$

by succesive conditioning on the history (natural filtration) $\mathcal{F}_i=\mathcal{F}(t_i)$ of the process N_{mRNA} up to times t_i, $i=n-1,n-2,\ldots,1$. In particular, $\mathcal{F}_i$ carries the information of the value of the process N_{mRNA} at t_i and its integral up to that time.

The evaluation of a general nested integral may seem a daunting task at first: The innermost conditional expectation in Eq. (5), $\mathbb{E}\left[e^{-A_n}|\mathcal{F}_{n-1}\right]$, yields a function of $N_{mRNA}(t_{n-1})$, which has to be multiplied by the factor $e^{-A_{n-1}}$ on the second line before calculating the next conditional expectation. Working outward (backward in history) to a general conditioning index i could therefore easily result in a very complex function of the unknown random variables $N_{mRNA}(t_i)$ and $\int_0^{t_i}N_{mRNA}(s)\,ds$. This may finally lead to a completely unmanageable expression. Next we show that the process of taking successive conditional expectations is stable in a sense that, in all stages, it is sufficient to be able to compute just a slightly more general integral than the innermost conditional expectation in Eq. (5), namely of the form $\mathbb{E}\left[z^{N_{mRNA}(t_i)}e^{-A_i}|\mathcal{F}_{i-1}\right]$.

We proceed by constructing the Kolmogorov forward equations for the joint process $\{N_{mRNA}(t),\int_0^t N_{mRNA}(s):t\geq 0\}$ as discussed in [3–5]. Let

$$p_j(u,t)=\mathbb{P}\left(N_{mRNA}(t)=j,\int_0^t N_{mRNA}(s)\,ds=u\,\Big|\,N_{mRNA}(0)=n_0\right). \tag{6}$$

Then

$$\frac{\partial p_j}{\partial t}(u,t)+j\frac{\partial p_j}{\partial u}(u,t)=a_{\mathrm{TF}}p_{j-1}(u,t)+\gamma_{\mathrm{m}}(j+1)p_{j+1}(u,t)-(a_{\mathrm{TF}}+\gamma_{\mathrm{m}}j)\,p_j(u,t) \tag{7}$$

with $p_j(u,0)=\delta_{j,n_0}\delta(u)$, and $p_j(u,t)=0$ for $j<0$ or $u<0$. Introducing the generating function with respect to j,

$$\pi(z,u,t)=\sum_{j=0}^{\infty}z^j p_j(u,t)$$

and a further Laplace transform with respect to u,

$$\tilde{\pi}(z,w,t)=\int_{[0,\infty)}e^{-wu}\pi(z,u,t)\,du\,,$$

we get

$$\frac{\partial\tilde{\pi}}{\partial t} + (zw - \gamma_{\rm m}(1-z))\,\frac{\partial\tilde{\pi}}{\partial z} = -a_{\rm TF}(1-z)\tilde{\pi} \tag{8}$$

subject to initial condition $\tilde{\pi}(z,w,0) = z^{n_0}$. This linear partial differential equation can be solved using the method of characteristics, and the result is

$$\tilde{\pi}(z,w,t) = \mathbb{E}\left[z^{N_{mRNA}(t)} e^{-w\int_0^t N_{mRNA}(s)\,ds} \middle| N_{mRNA}(0) = n_0\right] = \beta^{n_0}\exp\left[\frac{a_{\rm TF}}{\gamma_{\rm m}+w}\,(z-\beta-wt)\right] \tag{9}$$

where we have introduced

$$\beta = \beta(z,w,t) = \frac{\gamma_{\rm m}}{\gamma_{\rm m}+w}\left(1-e^{-(\gamma_{\rm m}+w)t}\right) + ze^{-(\gamma_{\rm m}+w)t}\,. \tag{10}$$

Now we can compute the innermost conditional expectation on the last line of Eq. (5) by setting $n_0 = N_{mRNA}(t_{n-1})$, $z = z_n = 1$ and $w = w_n = v_{\rm TF}F_{n-1}$, and by substituting $\Delta t_n = t_n - t_{n-1}$ as the time variable, in Eqs. (9,10). More precisely, we get

$$\mathbb{E}\left[e^{-A_n}|\mathcal{F}_{n-1}\right] = \beta_n^{N_{mRNA}(t_{n-1})} e^{\frac{a_{\rm TF}}{\gamma_{\rm m}+w_n}(z_n-\beta_n-w_n\Delta t_n)}\,, \tag{11}$$

where $\beta_n = \beta(z_n, w_n, \Delta t_n)$. Substituting this result in the next conditional expectation in Eq.(5) and applying formulae (9,10) again yields

$$\mathbb{E}\left[e^{-A_{n-1}}\mathbb{E}\left[e^{-A_n}|\mathcal{F}_{n-1}\right]|\mathcal{F}_{n-2}\right] = e^{\frac{a_{\rm TF}}{\gamma_{\rm m}+w_n}(z_n-\beta_n-w_n\Delta t_n)}\mathbb{E}\left[\beta_n^{N_{mRNA}(t_{n-1})}e^{-A_{n-1}}|\mathcal{F}_{n-2}\right] \tag{12}$$

$$= \beta_{n-1}^{N_{mRNA}(t_{n-2})} e^{\frac{a_{\rm TF}}{\gamma_{\rm m}+w_n}(z_n-\beta_n-w_n\Delta t_n)+\frac{a_{\rm TF}}{\gamma_{\rm m}+w_{n-1}}\left(z_{n-1}-\beta_{n-1}-w_{n_1}\Delta t_n\right)} \tag{13}$$

where $\beta_{n-1} = \beta(z_{n-1}, w_{n-1}, \Delta t_{n-1}) = \beta(\beta_n, v_{\rm TF}F_{n-2}, \Delta t_{n-1})$ because $z_{n-1} = \beta_n$, $w_{n-1} = v_{\rm TF}F_{n-2}$. By induction, it follows that the complete nested integral in Eq. (5) equals

$$\mathbb{E}\exp\left(-\sum_{i=1}^{n} A_i\right) = \mathbb{E}\,\beta_1^{N_{mRNA}(0)}\exp\left[\sum_{i=1}^{n}\frac{a_{\rm TF}}{\gamma_{\rm m}+v_{\rm TF}F_{i-1}}\left(\beta_{i+1}-\beta_i-v_{\rm TF}F_{i-1}\Delta t_i\right)\right] \tag{14}$$

$$= \exp\left[\sum_{i=1}^{n}\frac{a_{\rm TF}}{\gamma_{\rm m}+v_{\rm TF}F_{i-1}}\left(\beta_{i+1}-\beta_i-v_{\rm TF}F_{i-1}\Delta t_i\right)\right]\,, \tag{15}$$

where the last equality follows from the initial condition $N_{mRNA}(0) = 0$. The sequence $(\beta_i)_{i=1}^n$ satisfies the linear recursion

$$\beta_i = \beta(\beta_{i+1}, v_{\rm TF}F_{i-1}, \Delta t_i) = \frac{\gamma_{\rm m}}{\gamma_{\rm m}+v_{\rm TF}F_{i-1}}\left(1-e^{-(\gamma_{\rm m}+v_{\rm TF}F_{i-1})\Delta t_i}\right) + \beta_{i+1}e^{-(\gamma_{\rm m}+v_{\rm TF}F_{i-1})\Delta t_i} \tag{16}$$

with final condition $\beta_{n+1} = 1$. Since this is a linear non-homogeneous recursion, it can be solved by standard methods, yielding

$$\beta_i = \left[1+\frac{\gamma_{\rm m}}{\gamma_{\rm m}+v_{\rm TF}F_{n-1}}\left(e^{(\gamma_{\rm m}+v_{\rm TF}F_{n-1})\Delta t_n}-1\right)\right]e^{-\sum_{j=i}^{n}(\gamma_{\rm m}+v_{\rm TF}F_{j-1})\Delta t_j} \tag{17}$$

$$+\sum_{k=i}^{n-1}\frac{\gamma_{\rm m}}{\gamma_{\rm m}+v_{\rm TF}F_{k-1}}\left(e^{-\sum_{j=i}^{k-1}(\gamma_{\rm m}+v_{\rm TF}F_{j-1})\Delta t_j}-e^{-\sum_{j=i}^{k}(\gamma_{\rm m}+v_{\rm TF}F_{j-1})\Delta t_j}\right)\,. \tag{18}$$

Eqs. (14) and (17) provide a discrete approximation to our path integral problem. However, the solutions to diffusion equations are, in general, continuous, so we extend our analysis by taking the limit $n \to \infty$ while keeping t fixed. Stationary regulation can be later on studied by sending $t \to \infty$. The order of limits is important here. We first notice from the recursion relation (16) that

$$\beta_i = \gamma_{\rm m}\Delta t_i + \beta_{i+1}\left[1-(\gamma_{\rm m}+v_{\rm TF}F_{i-1})\Delta t_i\right] + \mathcal{O}(\Delta t_i^2)\,, \tag{19}$$

so the summand in Eq. (14) is approximated by

$$\beta_{i+1} - \beta_i - (\gamma_{\rm m}+v_{\rm TF}F_{i-1})\Delta t_i = (\beta_{i+1}-1)\,(\gamma_{\rm m}+v_{\rm TF}F_{i-1})\Delta t_i + \mathcal{O}(\Delta t_i^2)\,. \tag{20}$$

Hence the sum in Eq. (14) converges as

$$\mathbb{E}\exp\left(-\sum_{i=1}^{n} A_i\right) \to \exp\left[a_{\mathrm{TF}}\int_0^t (\beta(\lambda,s,t)-1)\ ds\right] \tag{21}$$

as $n\to\infty$ provided that the sequence $(\beta_i)_{i=1}^n$ itself converges to a function $\beta(\lambda,s,t)$ for each $t_i = it/n \to s$. From the explicit solution (17) for β_i, it can be seen that

$$\beta_i = e^{-\int_{t_i}^{t}(\gamma_{\mathrm{m}}+v_{\mathrm{TF}}F(\lambda,t-\tau))\,d\tau} + \gamma_{\mathrm{m}}\int_{t_i}^{t} e^{-\int_{t_i}^{\tau'}(\gamma_{\mathrm{m}}+v_{\mathrm{TF}}F(\lambda,t-\tau))\,d\tau}\,d\tau' + \mathcal{O}(\Delta t_i^2)\,, \tag{22}$$

so that we get our final result

$$\mathbb{E}\exp\left(-v_{\mathrm{TF}}\int_0^t N_{mRNA}(s)F(\lambda,t-s)ds\right) = \exp\left[a_{\mathrm{TF}}\int_0^t (\beta(\lambda,s,t)-1)\ ds\right]\ , \tag{23}$$

where

$$\beta(\lambda,s,t) = \exp\left[-\int_s^t (\gamma_{\mathrm{m}}+v_{\mathrm{TF}}F(\lambda,t-\tau))\ d\tau\right] + \gamma_{\mathrm{m}}\int_s^t \exp\left[-\int_s^{\tau'} (\gamma_{\mathrm{m}}+v_{\mathrm{TF}}F(\lambda,t-\tau))\ d\tau\right]\ d\tau'\,. \tag{24}$$

II. SI APPENDIX 2: LIST OF PARAMETERS FOR GILLESPIE ALGORITHM

In the simulation of enzyme catalyzed reaction, the substrate molecules were produced at rate $10/bs^{-1}$, where b is the burst size, so the total rate of production equals 10 molecules per second. In particular, the substrate molecules are just brought to the cell at this rate, and the process does not include the steps of gene expression, such as mRNA or protein maturation. The reaction volume was the same as used in the gene regulation simulations (see below). The parameters of MM kinetics were $k_1 = 0.185\,s^{-1}nM^{-1}$, $k_{-1} = 10\,s^{-1}$ and $k_2 = 20\,s^{-1}$.

The parameters for the gene regulation model were as given below. The reaction volume Ω (*i,e.* the volume surrounding the DNA, from within binding nonspecifically to the DNA is possible) was chosen to be a tube of length ℓ_Ω and radius R_Ω. The total, nonspecific binding rate is therefore $k_1^{ns} \times$ (No. TF molecules in the cell) $\times V_\Omega/V_{Cell}$. For comparison, the dimensions of *lac* repressor molecule have been measured 14 nm by 6 nm by 4.5 nm [6], the diameter of double-stranded is 1.2 nm, and a typical sliding distance along the DNA is of the order 50 bp [7, 8]. The length of the reaction volume given below corresponds to double of the typical sliding distance in nanometers.

Quantity	Symbol	Value
Transription rate	a_{TF}	$1/120\,s^{-1}$
Translation rate	v_{TF}	$1/120\,s^{-1}$
Mean protein maturation delay	Δ	$60\,s$
Number of protein maturation steps	κ	1
mRNA degradation rate	η_m	$1/120\,s^{-1}$
Protein (TF) degradation and dilution rate	η_P	$1/1200\,s^{-1}$
Length of reaction volume	ℓ_Ω	$34\,nm$
Reaction volume radius	R_Ω	$17\,nm$
Reaction volume $= \pi R_\Omega^2 \ell_\Omega$	V_Ω	$30870\,nm^3$
Cell volume	V_{Cell}	$1\,\mu m^3$
Binding rate to the DNA ($2000s^{-1}$ per particle in V_Ω)	k_1^{ns}	$0.037\,s^{-1}nM^{-1}$
Unbinding rate from the DNA (from nonspecific state)	k_{-1}^{ns}	$20\,s^{-1}$
Rate from nonspecific to specific binding state	k_1^{sp}	$2000\,s^{-1}$
Rate specific to nonspecific binding state	k_{-1}^{sp}	$10\,s^{-1}$

III. SI APPENDIX 3: EFFECTS OF TRANSCRIPTION AND TRANSLATION RATES OF THE TF, MULTIPLE BINDING SITES, AND CELL DIVISION

In this Appendix, we provide further evidence for the accuracy of the VCMME. First we show that the VCMME performs well in the gene regulation problem even if the rate transcription and translation rates are varied. The simulation results presented in the main article are based on transcription and translation rates that lead to very low TF concentrations (10 nM) in the cell, while typical concentrations in bacteria are in the range 10 nM − 10 μM. The stochastic effects will be small at micromolar concentrations. However, as Fig. 2 shows, they can still be quite large at and above 50 nM, perhaps even at a few hundred nM, if transcription rate is sufficiently low as in the left column of the figure. The translation rates corresponding to these slightly higher concentrations coincide with the ones measured in *E. coli* [9].

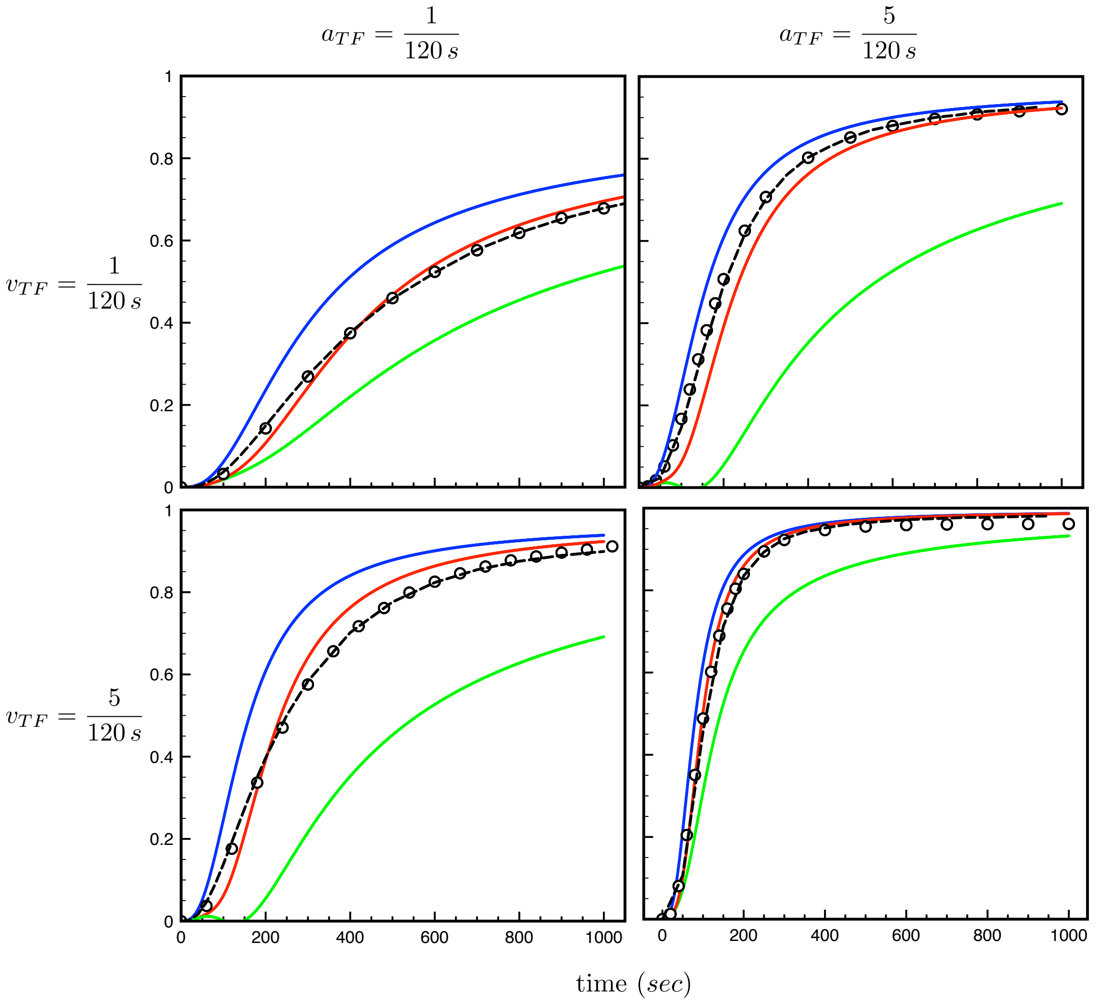

FIG. 1: Effect of TF transcription and translation rate to occupancy of the target site. The y-axis of each figure shows the occupation probability of the operator. Figures on one row have the same translation rate v_{TF} indicated on the left of the figure, and the figures on one column have the same transcription rate, which is written above that column. Upper left panel depicts the same data as in Fig. **3** of the main text but on a shorter time scale. The colour coding is the same as in the main text: blue solid curves show the MM equation, green solid curves the optimal lower bound, and the red solid curves the VCMME. Circles are the simulation data, and the blue dashed curves are the solutions of the analytical model.

Fig. 2 below shows that the the VCMME is able to predict the binding probability at the operator even if there are multiple binding sites for the TF on the bacterial chromosome. As the additional binding sites momentarily capture some of the TF molecules and they therefore act as a reservoir (the situation akin to a grand canonical ensemble of statistical mechanics), the total binding probability at one, specific site is lower than in the single binding site scenario given that the total TF synthesis level remains constant. This mechanism makes the number of TFs available for binding at the specific site smaller, in that the effective concentration is also lower, and therefore the mechanism can,

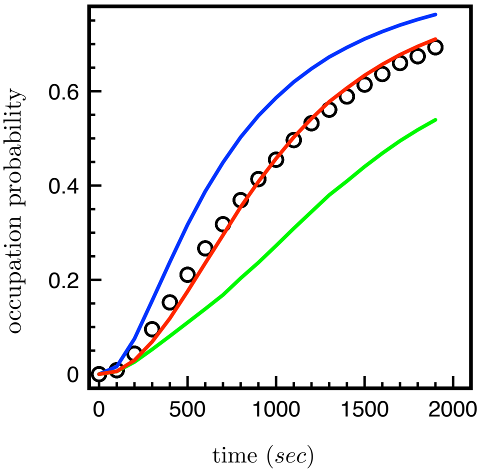

FIG. 2: Multiple binding sites (ten in this figure) act as a reservoir for TFs. The parameters are otherwise the same as in the upper left panel of Fig. 1 .The TF molecules bound at the alternative binding sites are incapable of contributing to the occupation of the operator, and the occupation probability of that specific site therefore increases more slowly than in the case of a single binding site. Otherwise, the results are analogous, and the VCMME quite accurately approximates the simulation data. The colour coding is the same as above and in the main text.

in principle, increase the relative concentration fluctuations. Yet if the synthesis level is increased to compensate for the concentration loss of the free TF, the concentration fluctuations most likely get smaller—at least after the initial transient. These speculations clearly require a separate study, especially because chromosomal structure and non-homogeneous diffusion in structured cells are also important factors in binding at multiple sites.

Finally, we would like to point out that simulation results from a model with cell division events included agree with our analytical model even though it does not incorporate actual cell division but a constant dilution of TF molecules due to cell growth instead. Fig. 3 shows the convergence towards the analytical result as the number of time traces (number of experiments with data from a single cell) increases from 100 to 500.

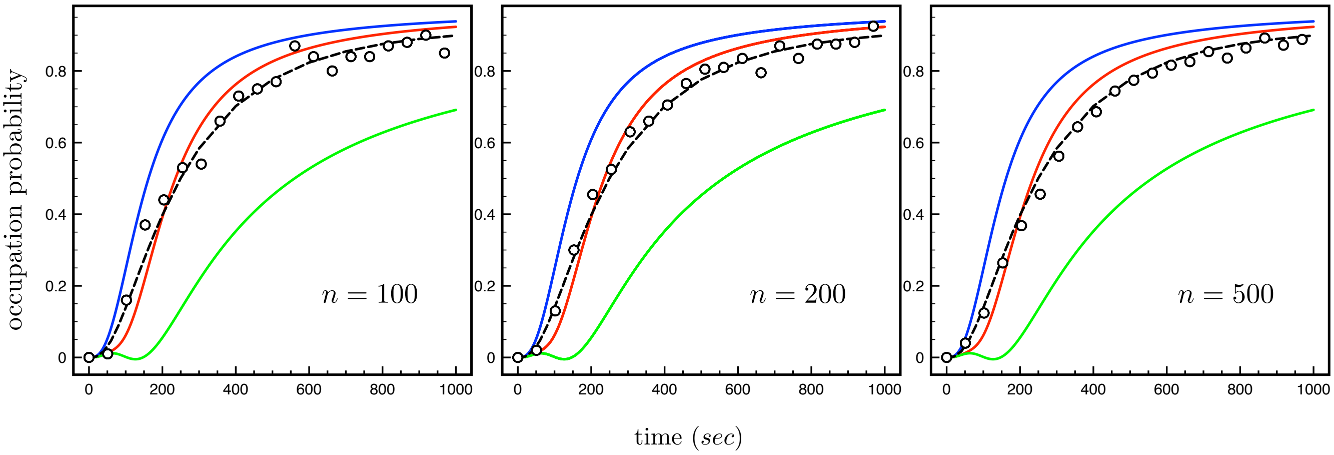

FIG. 3: Stochasticity in TF distribution upon cell division. In this simulation, the cells do not grow and only divide with the same rate as used for protein dilution in the other simulations for gene regulation. The TF molecules are distributed binomially to the daughter cells after each division event. The three panels show that the stochasticity averages out fairly quickly as more and more data (n denotes the number of time traces from individual cells) from single cells is gathered. The final panel with 500 traces suggests that our theoretical model (dashed line) is accurate even though it does not incorporate actual cell division events, but a constant rate dilution instead. The parameters of the simulation coincide with those of the lower left panel of Fig. 1.

[1] Billingsley, P. *Convergence of Probability Measures* (Wiley, New York, 1968).

[2] Picard, P. On the integral functionals of linear birth death immigration processes. *Statist. Decisions Suppl.* **2**, 105–109 (1985).

[3] Kendall, D.G. On the Generalized "Birth-and-Death" Process. *Ann. Math. Stat.* **19**, 1–15 (1948).

[4] Gani, J. & McNeil, D.R. Joint Distributions of Random Variables and Their Integrals for Certain Birth-Death and Diffusion Processes. *Adv. Appl. Prob.* **3**, 339–352 (1971).

[5] Gani, J. & Swift, R.J. A Simple Approach to the Integrals Under Three Stochastic Processes. *J. Stat. Theory Pract.* **2**, 559–568 (2008).

[6] Steitz, T.A., Richmond, T.J., Wise, D. & Engelman, D. The lac Repressor Protein: Molecular Shape, Subunit Structure, and Proposed Model for Operator Interaction Based on Structural Studies of Microcrystals. *Proc. Natl. Acad. Sci. USA* **71**, 593-597 (1971).

[7] Gowers, D.M., Wilson, G.G. & Halford, S.E. Measurement of the contributions of 1D and 3D pathways to the translocation of a protein along DNA. *Proc. Natl. Acad. Sci. USA* **102**, 15883–15888 (2005).

[8] van Zon, J.S., Morelli, M.J., Tănase-Nicola, S. & ten Wolde, P.R. Diffusion of Transcription Factors Can Drastically Enhance the Noise in Gene Expression. *Biophys. J.* **91**, 4350–4367 (2006).

[9] Cai, L., Friedman, N. & Xie, X.S. Stochastic protein expression in individual cells at the single molecule level. *Nature* **440**, 358–362 (2006).
